# Psychological distress, subjective well-being, and university dropout intention: a structural equation model examining indirect statistical associations involving academic burnout and satisfaction with education

**DOI:** 10.1186/s40359-026-05123-y

**Published:** 2026-07-06

**Authors:** Carlos Alberto Henao Periañez, Cruz Deicy Jaramillo-Bolívar, Marcio Alexander Castillo-Díaz

**Affiliations:** 1 Facultad de Salud, Escuela de Enfermería, Universidad del Valle, Cali, Valle del Cauca, Colombia; 2 Facultad de Ciencias de la Salud, Programa de Enfermería, Universidad Libre, Cali, Valle del Cauca Colombia; 3 Departamento de Psicología/Maestría en Psicometría y Evaluación Educativa, Facultad de Ciencias Sociales, Universidad Nacional Autónoma de Honduras, Tegucigalpa, Honduras

**Keywords:** Psychological distress, Subjective well-being, University dropout intention, Structural equation model, Undergraduate students, Academic burnout, Satisfaction with education

## Abstract

**Background:**

Psychological distress and subjective well-being are increasingly recognized as related but distinct dimensions of mental health that may influence academic outcomes in higher education. Consistent with the dual-continuum perspective of mental health, few studies have simultaneously examined how these dimensions relate to academic experiences and university dropout intention within a single analytical framework. This study aimed to analyze the relationships among psychological distress, subjective well-being, and university dropout intention, while examining indirect statistical associations involving academic burnout and satisfaction with education within the proposed model.

**Methods:**

A cross-sectional study was conducted with 614 undergraduate students from a private university in Cali, Colombia, selected through proportional stratified sampling. Data were collected using the Depression, Anxiety, and Stress Scale (DASS-21), the WHO-5 Well-Being Index, and the Screening Instrument for Students At-Risk of Dropping Out. Structural equation modeling (SEM) with a weighted least squares mean and variance adjusted (WLSMV) estimator was used to test direct and indirect relationships. Model fit was assessed using CFI, TLI, RMSEA, and SRMR.

**Results:**

The final model showed good fit (CFI = 0.998; TLI = 0.998; RMSEA = 0.044; SRMR = 0.051) and explained a substantial proportion of variance in university dropout intention (R² = 0.764). Academic burnout was strongly associated with university dropout intention (β = 0.900, *p* < .001), while satisfaction with education showed a negative association (β = −0.175, *p* < .001). Psychological distress was positively associated with academic burnout (β = 0.322, *p* < .001), and subjective well-being was negatively associated (β = −0.117, *p* = .011). No significant direct associations were found between mental health variables and university dropout intention; however, academic burnout emerged as the principal academic-experience correlate associated with dropout intention and was involved in the indirect statistical associations observed between mental health indicators and university dropout intention. Indirect associations involving satisfaction with education were not statistically significant.

**Conclusion:**

Academic burnout emerged as the primary academic correlate associated with university dropout intention within the proposed model. By simultaneously considering psychological distress and subjective well-being, this study extends the application of the dual-continuum perspective of mental health to the context of university dropout intention. These findings highlight the relevance of considering both dimensions of mental health alongside academic experiences when examining university dropout intention. Because of the cross-sectional design, the observed relationships should be interpreted as statistical associations rather than causal effects and warrant further investigation in longitudinal studies.

## Introduction

University dropout remains a major challenge for higher education systems worldwide because of its academic, economic, and social consequences. Leaving university before degree completion has been associated with reduced opportunities for human capital development, lower employability, and inefficiencies in the allocation of institutional and public resources [1, 2]. Although university dropout is a multifactorial phenomenon influenced by individual, academic, institutional, and socioeconomic factors, university dropout intention has gained increasing attention because it represents one of the most proximal cognitive antecedents of actual dropout behavior [3, 4]. Therefore, understanding the factors associated with students’ intentions to leave higher education is essential for informing persistence-oriented policies and support strategies.

Among the factors associated with persistence-related processes, mental health has emerged as an important explanatory domain. Psychological distress, commonly conceptualized through symptoms of depression, anxiety, and stress [5, 6], is highly prevalent among university students worldwide and has been linked to a range of adverse academic outcomes [7, 8]. Previous studies have reported associations between psychological distress and poorer academic adjustment, lower engagement, and increased consideration of leaving university programs [9, 10]. However, much of the existing literature has predominantly focused on negative indicators of mental health, potentially overlooking the role of positive dimensions of psychological functioning in understanding students’ academic trajectories.

Subjective well-being constitutes one such positive dimension and reflects individuals’ evaluations of their overall psychological functioning and life satisfaction [11]. Evidence suggests that higher levels of well-being are associated with favorable educational outcomes, including better adjustment to university life and greater academic persistence [12–14]. From a theoretical perspective, the simultaneous examination of psychological distress and subjective well-being is supported by the dual-continuum model of mental health [15]. According to this framework, the absence of psychological symptoms does not necessarily imply the presence of positive well-being, as these dimensions represent related but distinct aspects of mental health. Consequently, assessing only psychological distress may provide an incomplete understanding of students’ psychological functioning and its associations with persistence-related outcomes.

The specific contribution of the dual-continuum perspective in the present study lies in examining whether negative and positive dimensions of mental health exhibit distinct patterns of association with academic experiences and university dropout intention within the same explanatory framework. This approach may offer a more comprehensive understanding of the psychological factors associated with persistence in higher education.

Mental health indicators may influence university dropout intention not only directly but also through their associations with students’ academic experiences. Academic burnout, characterized by emotional exhaustion, cynicism toward academic activities, and reduced academic efficacy, has been increasingly recognized as a relevant factor in higher education contexts [16]. Previous studies have documented associations between psychological distress and academic burnout, suggesting that students experiencing elevated emotional symptoms may perceive greater academic overload and diminished coping resources [17, 18]. In turn, academic burnout has consistently been associated with lower academic engagement, poorer educational experiences, and stronger intentions to discontinue university studies [18, 19].

Another academic-experience variable that may be relevant to persistence-related processes is satisfaction with education. Satisfaction with education reflects students’ evaluations of the quality, value, and overall experience of their academic programs and has been associated with institutional commitment and persistence [20]. Students reporting lower educational satisfaction appear to be more likely to consider abandoning their studies [21]. However, compared with academic burnout, empirical evidence supporting satisfaction with education as a mechanism linking psychological functioning to university dropout intention remains limited. Therefore, its role within the present model should be interpreted as exploratory, while remaining theoretically grounded in persistence frameworks emphasizing students’ evaluations of their educational experiences [22–24].

Recent studies have highlighted the interplay between mental health and academic experiences in explaining university dropout intention. For example, network analyses have shown that academic burnout, depression, anxiety, and dropout intention are closely interconnected [18]. Similarly, structural models have identified burnout and satisfaction-related constructs as relevant factors associated with persistence-related outcomes [25, 26]. Nevertheless, important gaps remain in the literature. First, relatively few studies have simultaneously examined psychological distress, subjective well-being, academic burnout, satisfaction with education, and university dropout intention within a single analytical framework. Second, previous studies have rarely incorporated the dual-continuum perspective of mental health when investigating persistence-related outcomes. Third, although burnout and educational satisfaction have been linked to dropout intention, their potential roles as intermediary academic-experience pathways have received comparatively less attention.

Unlike previous Structural equation modeling (SEM) studies that have examined mental health symptoms or academic experiences separately, the present study integrates indicators of psychological distress and subjective well-being within the dual-continuum framework while simultaneously examining academic burnout and satisfaction with education in relation to university dropout intention. By distinguishing between mental health indicators and academic experiences, the proposed model seeks to clarify how these domains may operate together in explaining students’ intentions to leave higher education.

The Colombian context provides an important setting for examining these relationships. Latin American countries continue to face substantial socioeconomic inequalities that may shape students’ access to resources, academic experiences, and persistence trajectories [1]. In Colombia, university dropout remains a significant concern, and evidence integrating mental health and academic-experience variables within explanatory models of dropout intention remains scarce. Furthermore, private universities often enroll students from diverse socioeconomic backgrounds who may encounter unique financial, academic, and psychosocial challenges that influence persistence-related processes. Consequently, context-specific evidence may contribute to the development of targeted institutional strategies aimed at supporting student retention and well-being.

Understanding the relationships among mental health indicators, academic experiences, and university dropout intention may contribute to a more comprehensive perspective on persistence processes in higher education. Therefore, the present study aimed to analyze the relationships among psychological distress, subjective well-being, and university dropout intention while examining the indirect statistical associations involving academic burnout and satisfaction with education within a structural equation modeling framework. The novelty of this study lies in integrating the dual-continuum perspective of mental health with an explanatory model that distinguishes psychological functioning from academic experiences, thereby extending previous SEM approaches to university dropout intention.

**Fig. 1:**
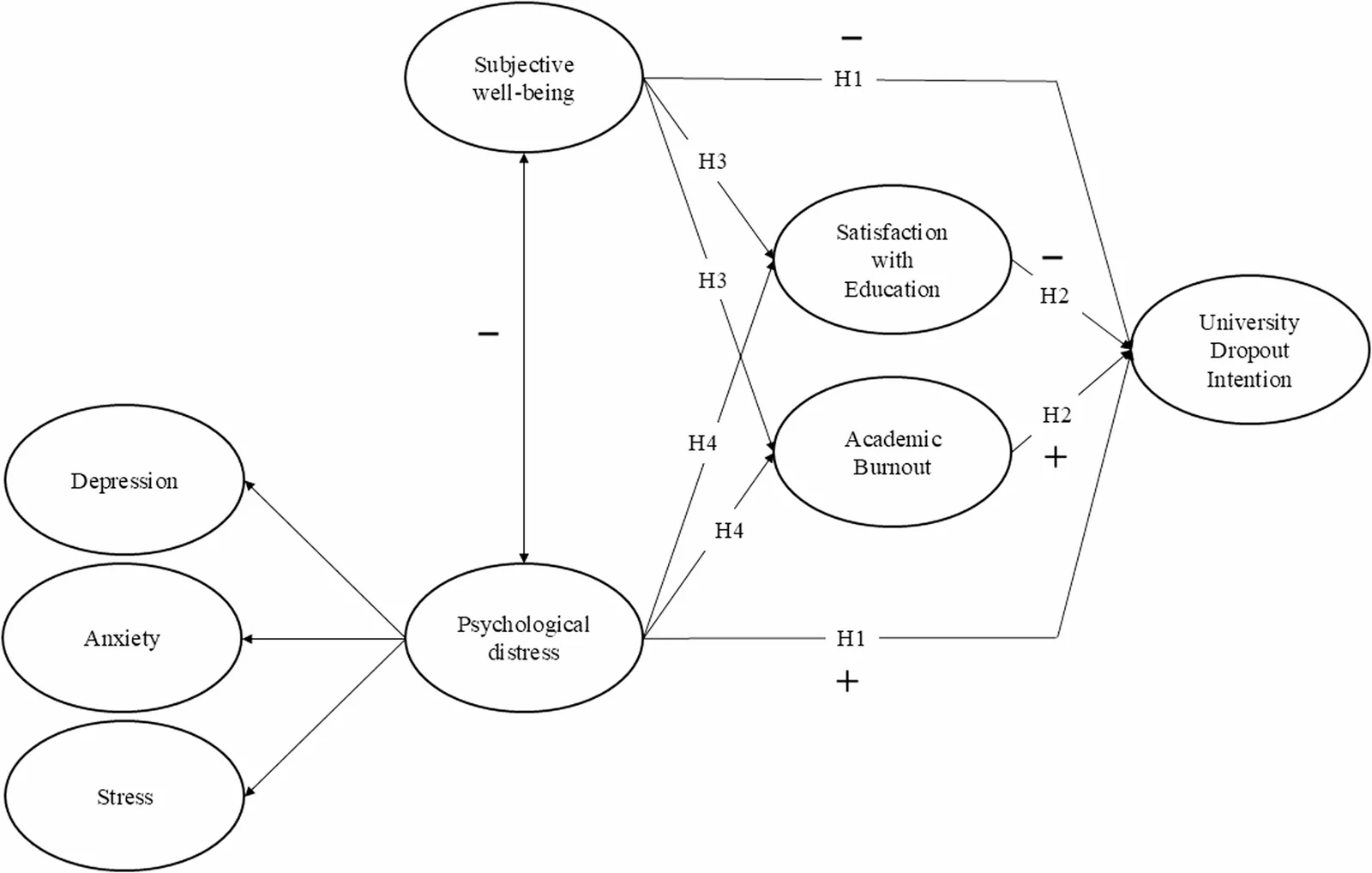
Hypothesized structural model of the relationships among psychological distress, subjective well-being, academic burnout, satisfaction with education, and university dropout intention. *Note*: Psychological distress is modeled as a latent variable represented by depression, anxiety, and stress. H1–H2: Direct associations on university dropout intention. H3–H4: Indirect associations via academic burnout and satisfaction with education (from subjective well-being and psychological distress, respectively). Signs (+/–) indicate the direction of the hypothesized relationships

Based on the theoretical framework and previous empirical evidence, several hypotheses were formulated and are presented in the hypothesized model (Fig. 1). First, psychological distress is expected to be positively associated with university dropout intention, whereas subjective well-being is expected to be negatively associated with university dropout intention (H1). Second, academic burnout is expected to be positively associated with university dropout intention, while satisfaction with education is expected to be negatively associated with it (H2). Third, academic burnout and satisfaction with education are expected to be involved in the indirect statistical associations between subjective well-being and university dropout intention (H3). Finally, these academic-experience variables are expected to be involved in the indirect statistical associations between psychological distress and university dropout intention (H4). The hypotheses were intentionally formulated at the conceptual-domain level, reflecting the integrated nature of the proposed structural model and facilitating the interpretation of relationships among mental health indicators, academic experiences, and university dropout intention.

The analysis of these mechanisms is particularly relevant given the broad social implications of higher education completion. Education plays a fundamental role in human capital development and in strengthening key sectors of society. Consequently, factors that undermine academic persistence may have repercussions not only for individuals but also for social and economic development. In this regard, evidence on the psychological and academic factors associated with university dropout intention may provide a valuable basis for the design of institutional and public policies aimed at promoting student mental health in relation to university dropout intention and related academic experiences.

## Methods

### Study design

A cross-sectional observational study was conducted during the 2025-1 and 2025-2 academic periods at a private university located in Cali, Colombia. The study design and reporting of results followed the recommendations of the STROBE (Strengthening the Reporting of Observational Studies in Epidemiology) guidelines for observational studies [27].

### Population, sample, and sampling

The study was carried out with undergraduate students enrolled in programs across different faculties of the institution, including Health Sciences, Law, Political and Social Sciences, Economic, Administrative and Accounting Sciences, and Engineering. During the 2025-1 academic period, the total population of undergraduate students enrolled in these faculties was 2,909, which constituted the target population of the study.

The required sample size was estimated using a priori power analysis for structural equation modeling. Assuming a small-to-moderate anticipated effect size (0.20), a statistical power of 0.80, and a significance level of 0.05, the specified model—including eight latent variables and 38 observed indicators—yielded a recommended minimum sample size of 444 participants to ensure adequate statistical power and model stability [28].

Participants were selected using proportional stratified sampling, with academic faculties serving as strata. The number of participants selected from each faculty was determined proportionally based on enrollment figures. Within each stratum, a list of eligible students was obtained from institutional records, and participants were selected using a simple random sampling procedure (computer-generated random numbers). Initially, 444 students were invited; subsequently, additional students were randomly selected and invited using the same procedure to compensate for non-response and preserve proportional representation across strata. Because participation was anonymous and additional invitations were issued to compensate for non-response, the total number of invited students was not tracked; therefore, an exact response rate could not be calculated.

### Inclusion and exclusion criteria

Eligible participants were undergraduate students aged 18 years or older, enrolled in programs within the participating faculties during the 2025-1 and 2025-2 academic periods, who voluntarily agreed to participate and provided informed consent prior to completing the questionnaire. Students who did not provide consent or who failed to complete the questionnaire in full, particularly regarding the main study variables, were excluded. No data imputation procedures were applied. Only those who met these criteria and completed the instrument were included in the final analytical sample.

### Instruments and data collection procedures

Data were collected using a structured, self-administered questionnaire delivered electronically via the Google Forms platform. This approach ensured anonymous and standardized data collection across all participating students. Selected students were invited to participate through institutional email communication and classroom announcements coordinated with faculty members.

Psychological distress was assessed using the Depression, Anxiety, and Stress Scale–21 (DASS-21), a 21-item self-report instrument designed to measure symptoms of psychological distress based on the tripartite model of depression, anxiety, and stress [6]. The scale comprises three dimensions, each consisting of seven items: depression, anxiety, and stress. Participants indicate how frequently they have experienced each symptom over the previous two weeks using a four-point Likert scale ranging from 0 (never) to 3 (almost always or most of the time). The Spanish-language version of the DASS-21 was used. Dimension scores were obtained by summing the responses to the seven items corresponding to each subscale, with higher scores indicating greater psychological distress. Recent studies have reported robust evidence of validity, reliability, and factorial invariance of the DASS-21 among Latin American university students, with adequate levels of internal consistency for its dimensions: depression (α = 0.88; ω = 0.88), anxiety (α = 0.82; ω = 0.83), and stress (α = 0.90; ω = 0.90) [29].

Subjective well-being was assessed using the World Health Organization Well-Being Index (WHO-5), a brief instrument developed by the World Health Organization to measure subjective well-being [30]. The instrument consists of five self-report items that assess the frequency of positive affective experiences during the previous two weeks. Each item is rated on a six-point Likert scale ranging from 0 (at no time) to 5 (all of the time). The Spanish-language version of the WHO-5 was used. Total scores were obtained by summing the responses across the five items, with higher scores indicating greater subjective well-being. This scale has been widely used in both clinical and population-based studies and is supported by empirical evidence across diverse populations and cultural contexts [31]. Additionally, studies conducted with university students in Latin America have reported adequate psychometric properties of the WHO-5, including satisfactory levels of internal consistency (α = 0.88; ω = 0.89) [32].

University dropout intention, satisfaction with education, and academic burnout were assessed using the Screening Instrument for Students At-Risk of Dropping Out [33], a self-report scale based on a multidimensional theoretical model of university dropout. The instrument comprises three dimensions: university dropout intention, satisfaction with education, and academic burnout, each consisting of four items. Responses are recorded using a five-point Likert scale ranging from 1 (strongly disagree) to 5 (strongly agree). The Spanish-language version of the instrument was used. Scores for each subscale were calculated by averaging the responses to the corresponding four items, with higher scores indicating higher levels of university dropout intention, satisfaction with education, and academic burnout, respectively. Previous studies have reported adequate evidence of validity and reliability for this scale among university students, including satisfactory levels of internal consistency across its subscales: academic burnout (α = 0.83; ω = 0.83), satisfaction with education (α = 0.81; ω = 0.80), and university dropout intention (α = 0.76; ω = 0.75) [33]. Recent research conducted with university students has also confirmed adequate internal consistency of the instrument in Latin American contexts [25].

Prior to completing the questionnaire, participants were presented with a digital informed consent form, and agreement to participate was required to access the survey. Participation was voluntary, anonymous, and confidential, and the estimated time to complete the questionnaire was approximately 15 to 20 min. Upon completion of the survey, students were provided with information about institutional academic and psychosocial support programs available at the university, aimed at promoting student well-being and offering support if needed.

### Ethical considerations

The study was approved by the Research Ethics and Bioethics Committee of Universidad Libre, Cali Campus, under Act No. CAL-131,202,409-CE, issued on November 8, 2024, within the framework of the project “Psychosocial determinants of well-being and quality of life in university students.” The ethical review was conducted in accordance with Resolution 008430 of 1993 issued by the Colombian Ministry of Health.

Prior to participation, students were informed about the objectives of the study and provided voluntary informed consent. Participation was anonymous and confidential, and participants were free to withdraw from the study at any time without academic consequences. No ethical issues were reported during data collection.

### Data analysis

Data analysis was conducted in three phases. First, descriptive statistics were calculated for all study variables. Categorical variables are presented as frequencies and percentages, while continuous variables are reported using means, standard deviations, and distributional indices (skewness and kurtosis). Common method bias was assessed using Harman’s single-factor test (EFA including all items), considering < 40% explained variance as acceptable [34].

Second, bivariate associations were examined. Given deviations from normality based on the Shapiro–Wilk test, Spearman’s rank correlation coefficient was used.

Third, hypotheses were tested using structural equation modeling (SEM), which integrates measurement and structural components [35]. Measurement models were previously evaluated using confirmatory factor analysis (CFA) at the item level. Reliability was assessed using ordinal alpha (α ≥ 0.70) and McDonald’s omega (ω ≥ 0.70) [36], and convergent validity using average variance extracted (AVE ≥ 0.50) [37]. Discriminant validity was assessed using the heterotrait–monotrait ratio of correlations (HTMT < 0.90) [38].

The structural model specified a multiple mediation framework in which factors were associated with university dropout intention directly and indirectly through satisfaction with education and academic burnout (Fig. 1). Given the cross-sectional design, the estimated indirect effects were interpreted as indirect statistical associations rather than evidence of causal mediation. Sociodemographic and academic variables (e.g., gender, GPA, employment status, and time at university) were described to characterize the sample but were not included in the primary SEM, which was specified to examine the hypothesized relationships among the study constructs. Models were estimated using the weighted least squares mean and variance adjusted (WLSMV) estimator, appropriate for ordinal data [35]. Model fit was evaluated using the comparative fit index (CFI) and Tucker–Lewis index (TLI) (≥ 0.95), and the root mean square error of approximation (RMSEA) and standardized root mean square residual (SRMR) (≤ 0.08) [39, 40].

Direct, indirect, and total effects were estimated using standardized coefficients (std.all), and coefficients of determination (R²) were reported for endogenous variables. 95% confidence intervals were calculated using the delta method [41]. Analyses were conducted in R (version 4.4.2) using RStudio, *lavaan* [42], and *semTools* [43] packages.

## Results

### Descriptive analyses

**Fig. 2:**
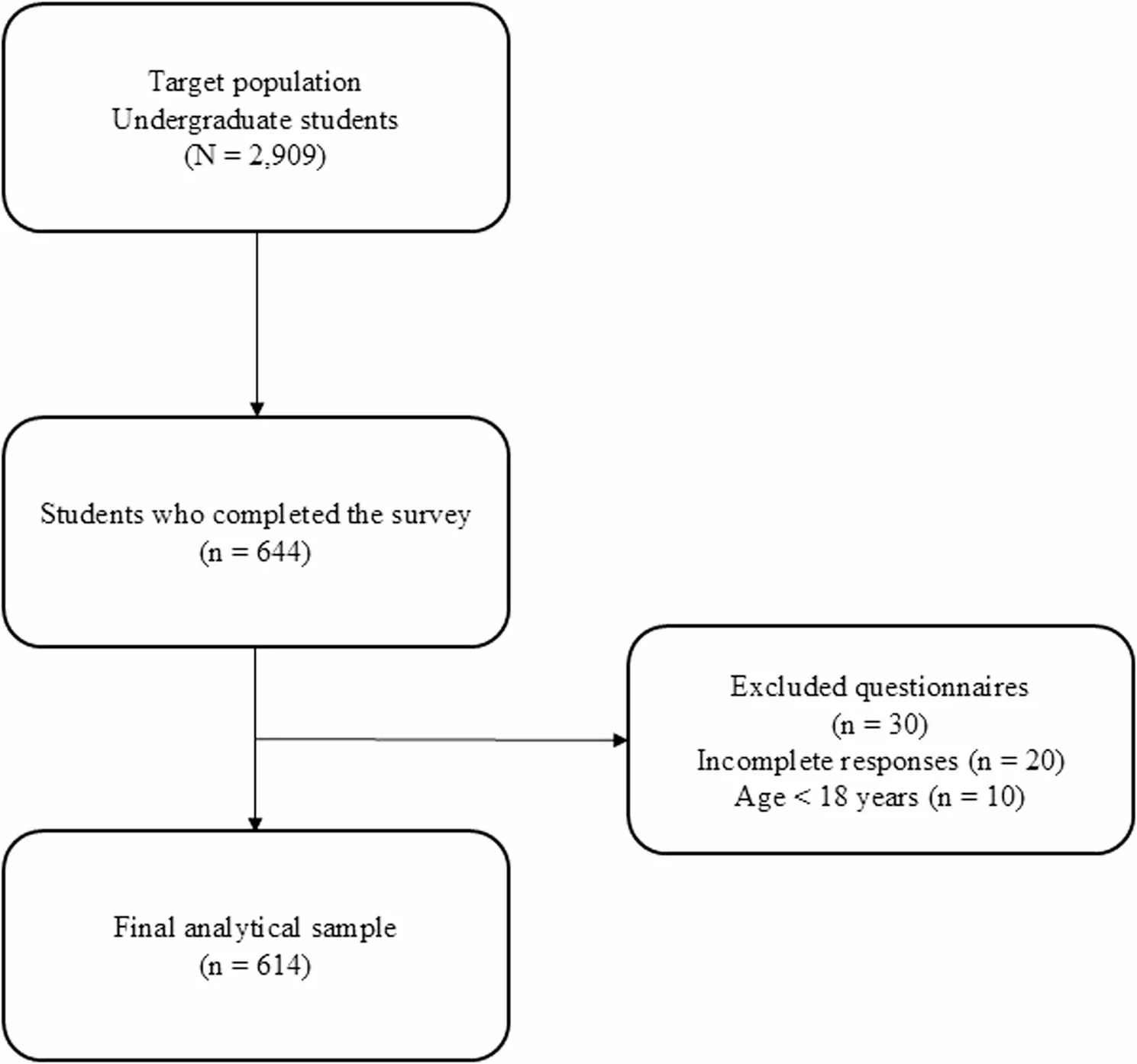
Flow diagram of participant recruitment, exclusions, and final analytical sample. Note: Additional invitations were issued to compensate for non-response; therefore, an exact response rate could not be calculated

A total of 644 students completed the survey following the initial and additional invitation procedures implemented to preserve proportional representation across strata. Of these, 20 questionnaires (3.1%) were excluded due to incomplete responses in the main study variables, and 10 participants (1.6%) were excluded because they were younger than 18 years. Consequently, the final analytical sample comprised 614 university students, representing 95.3% of completed questionnaires and 21.1% of the target population, who met all eligibility criteria (Fig. 2).

Participants had a mean age of 21.62 years (SD = 3.94) and were predominantly female (64.17%). Most students belonged to the middle socioeconomic stratum (57.98%) and reported living with their nuclear or extended family (76.22%). The distribution of participants across faculties reflected the stratified sampling design, ensuring representation of students from the main academic units of the university. Academically, the majority had been enrolled at the university for two years or more (56.35%) and reported not working while studying (68.24%). The detailed sociodemographic and academic characteristics of the participants are presented in Table 1.

**Table 1:** Sociodemographic and academic characteristics of the study participants (*n* = 614)

Variable	*n* (%) / Mean ± SD
Age (years)	21.62 ± 3.94
Range	18–47
Gender	
Female	394 (64.17)
Male	219 (35.67)
Non-binary	1 (0.16)
Ethnicity	
White/Mestizo	473 (77.04)
Afro-descendant / Afro-Colombian	119 (19.38)
Indigenous	22 (3.58)
Socioeconomic stratum*	
Low	163 (26.55)
Middle	356 (57.98)
High	95 (15.47)
Living arrangement	
With nuclear or extended family	468 (76.22)
Alone or with non-family members	146 (23.78)
Faculty	
Health Sciences	437 (71.17)
Law, Political and Social Sciences	88 (14.33)
Economic, Administrative and Accounting Sciences	67 (10.91)
Engineering	22 (3.58)
Time at university	
< 2 years	268 (43.65)
≥ 2 years	346 (56.35)
Employment status (study and work)	
No	419 (68.24)
Yes	195 (31.76)
Current grade point average (GPA)	
< 3.0	10 (1.63)
3.0–3.9	297 (48.37)
4.0–5.0	276 (44.95)
First semester (not applicable)	31 (5.05)

*M* Mean, *SD* Standard deviation, *n (%)* frequency and percentage, *GPA* Grade point average (range: 0.0–5.0)

* The Colombian residential stratification system is widely used as a proxy indicator of socioeconomic conditions

Harman’s single-factor test was conducted using an exploratory factor analysis including all items from the DASS-21, WHO-5, and the Screening Instrument for Students at Risk of Dropping Out. The single factor accounted for 37.8% of the total variance, below the 40% threshold, suggesting that common method bias is unlikely to affect the results. Table 2 presents the descriptive statistics of the study variables. The psychological distress indicators—depression, anxiety, stress, and the overall psychological distress score—showed distributions centered around moderate values, with comparable variability across dimensions. Subjective well-being was slightly skewed toward higher values, as was satisfaction with education, which showed a more pronounced concentration in the upper range. Academic burnout was observed at intermediate levels, whereas university dropout intention was concentrated toward lower values, indicating generally low intention to leave university.

**Table 2:** Descriptive statistics of the psychological and academic variables included in the study (*n* = 614)

Variable	M	SD	Min	Max	Skewness	Kurtosis
Psychological variables						
Depression	7.28	5.85	0	21	0.57	−0.55
Anxiety	7.18	5.92	0	21	0.56	−0.63
Stress	8.85	6.06	0	21	0.24	−0.84
Psychological distress	23.31	16.96	0	63	0.48	−0.59
Subjective well-being	16.55	5.64	0	25	−0.37	−0.44
Academic experience variables						
Academic burnout	9.45	4.28	4	20	0.57	−0.42
Satisfaction with education	14.43	5.53	4	20	−0.84	−0.65
Outcome variable						
University dropout intention	7.41	4.14	4	20	1.31	1.07

Depression, anxiety, and stress correspond to the three subscales of the Depression Anxiety Stress Scales (DASS-21)

Psychological distress represents the total score of the DASS-21

*M* mean, *SD* standard deviation

### Bivariate analyses

Table 3 presents the correlations among the study variables. Psychological distress was moderately and negatively associated with subjective well-being (ρ = −0.486, *p* < .001) and positively related to academic burnout (ρ = 0.342, *p* < .001) and university dropout intention (ρ = 0.194, *p* < .001). In turn, academic burnout showed a strong positive association with university dropout intention (ρ = 0.583, *p* < .001) and a weak negative correlation with subjective well-being (ρ = −0.262, *p* < .001). In contrast, satisfaction with education showed small and non-significant correlations with psychological distress (ρ = 0.058, *p* = .148) and subjective well-being (ρ = −0.042, *p* = .303), as well as a weak but statistically significant negative association with university dropout intention (ρ = −0.186, *p* < .001).

**Table 3:** Correlations among the psychological and academic variables included in the study (*n* = 614)

Variable	1	2	3	4	5
1. Psychological distress	1				
2. Subjective well-being	−0.486***	1			
3. Academic burnout	0.342***	−0.262***	1		
4. Satisfaction with education	0.058	−0.042	0.122**	1	
5. University dropout intention	0.194***	−0.126**	0.583***	−0.186***	1

Values correspond to Spearman’s rank correlation coefficients

Psychological distress represents the total score of the DASS-21

*p < .05, **p < .01, ***p < .001

### Structural equation modeling analysis

#### Measurement models

For the DASS-21, a second-order factor model was estimated, specifying depression, anxiety, and stress as first-order dimensions loading onto a higher-order psychological distress construct. The model demonstrated acceptable fit to the data (χ² = 418.250, df = 186; CFI = 0.999; TLI = 0.999; SRMR = 0.031; RMSEA = 0.045, 95% CI = 0.039–0.051). Standardized factor loadings were statistically significant (*p* < .001), ranging from λ = 0.649 to 0.894 for depression, λ = 0.706 to 0.901 for anxiety, and λ = 0.732 to 0.904 for stress. Loadings of the first-order factors onto the higher-order psychological distress construct ranged from λ = 0.958 to 0.981. Internal consistency coefficients were as follows: depression (ω = 0.922; α = 0.942), anxiety (ω = 0.925; α = 0.945), and stress (ω = 0.928; α = 0.946). AVE values ranged from 0.713 to 0.723. The higher-order factor showed Ω_ho_ = 0.981.

For the WHO-5, a one-factor model was estimated (χ² = 11.300, df = 4; CFI = 1.000; TLI = 1.000; SRMR = 0.012; RMSEA = 0.055, 95% CI = 0.018–0.093). Standardized factor loadings were statistically significant (*p* < .001), ranging from λ = 0.808 to 0.957. Internal consistency coefficients were ω = 0.932 and α = 0.955. The AVE was 0.804.

For the Screening Instrument for Students At-Risk of Dropping Out, a three-factor first-order model corresponding to satisfaction with education, academic burnout, university dropout intention and was estimated (χ² = 234.398, df = 50; CFI = 0.999; TLI = 0.999; SRMR = 0.064; RMSEA = 0.078, 95% CI = 0.068–0.088). Standardized factor loadings were statistically significant (*p* < .001), ranging from λ = 0.947 to 0.983 for satisfaction with education, λ = 0.700 to 0.915 for academic burnout, and λ = 0.812 to 0.963 for university dropout intention. Internal consistency was high across all dimensions: satisfaction with education (ω = 0.966; ordinal α = 0.982), academic burnout (ω = 0.837; ordinal α = 0.911), and university dropout intention (ω = 0.913; ordinal α = 0.945). AVE values were 0.932 for satisfaction with education, 0.691 for academic burnout, and 0.809 for university dropout intention. All HTMT coefficients were below the recommended threshold of 0.90, supporting adequate discriminant validity among the constructs. Specifically, the HTMT coefficients were 0.252 (satisfaction with education – academic burnout), 0.027 (satisfaction with education – university dropout intention), and 0.770 (academic burnout – university dropout intention).

#### Structural model

**Fig. 3:**
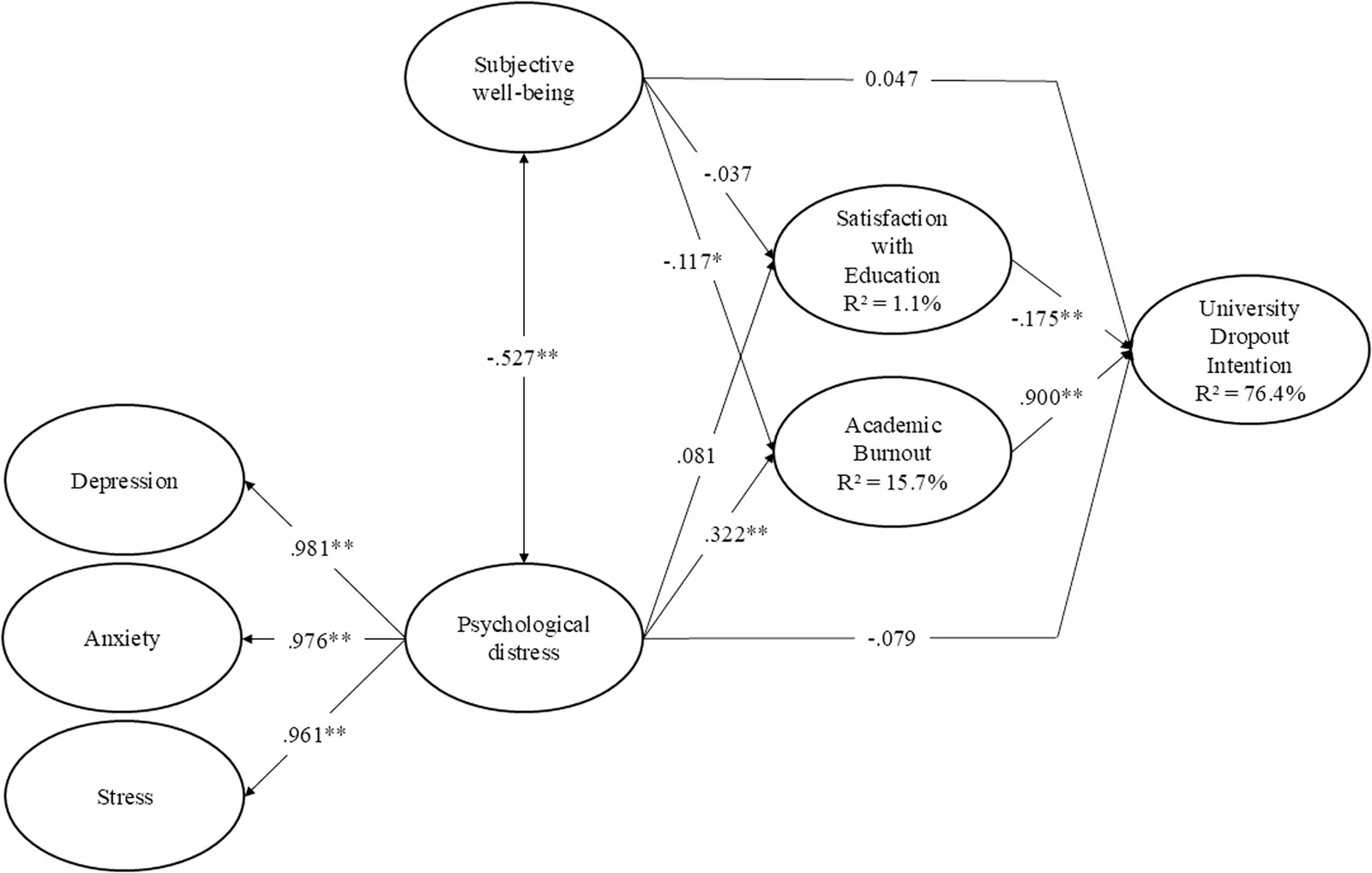
Structural equation model of psychological distress, subjective well-being, academic burnout, satisfaction with education, and university dropout intention. *Note*: Values represent standardized regression coefficients (β). R² values are shown for endogenous variables. *p < .05, **p < .001

The structural equation model showed good fit to the data (χ² = 1424.204, df = 651; CFI = 0.998; TLI = 0.998; SRMR = 0.051; RMSEA = 0.044 [95% CI = 0.041–0.047]). The model with standardized estimates and R² values is presented in Fig. 3. The explained variance (R²) was 0.011 for satisfaction with education, 0.157 for academic burnout, and 0.764 for university dropout intention.

Standardized direct associations are presented in Table 4. Psychological distress was positively associated with academic burnout (β = 0.322, *p* < .001), while subjective well-being was negatively associated with academic burnout (β = −0.117, *p* = .011). No statistically significant direct associations were observed between psychological distress and satisfaction with education (β = 0.081, *p* = .126) or university dropout intention (β = −0.079, *p* = .059), nor between subjective well-being and satisfaction with education (β = −0.037, *p* = .473) or university dropout intention (β = 0.047, *p* = .187). Satisfaction with education was negatively associated with university dropout intention (β = −0.175, *p* < .001), whereas academic burnout was positively associated with university dropout intention (β = 0.900, *p* < .001).

**Table 4:** Standardized direct associations of psychological distress and subjective well-being with satisfaction with education, academic burnout, and university dropout intention (*n* = 614)

Predictor	Outcome	β	95% CI	*p*
PD →	SE	0.081	−0.023, 0.185	0.126
SWB →	SE	−0.037	−0.138, 0.064	0.473
PD →	AB	0.322	0.224, 0.419	< 0.001
SWB →	AB	−0.117	−0.207, − 0.027	0.011
PD →	UDI	−0.079	−0.161, 0.003	0.059
SWB →	UDI	0.047	−0.023, 0.117	0.187
SE →	UDI	−0.175	−0.243, − 0.108	< 0.001
AB →	UDI	0.900	0.859, 0.940	< 0.001

*PD* psychological distress, *SWB* subjective well-being, *SE* satisfaction with education, *AB* academic burnout, *UDI* university dropout intention, β standardized regression coefficient, *CI* confidence interval

Standardized indirect and total associations are presented in Table 5. Psychological distress showed a significant indirect association on university dropout intention through academic burnout (β = 0.289, *p* < .001), whereas the indirect effect through satisfaction with education was not statistically significant (β = −0.014, *p* = .161). The total indirect associations of psychological distress on university dropout intention was significant (β = 0.275, *p* < .001), and the total associations was also statistically significant (β = 0.196, *p* = .001). For subjective well-being, the indirect effect through satisfaction with education was not statistically significant (β = 0.006, *p* = .472), while the indirect association through academic burnout was statistically significant (β = −0.105, *p* = .011). The total indirect association of subjective well-being on university dropout intention was significant (β = −0.099, *p* = .014), whereas the total associations was not statistically significant (β = −0.052, *p* = .311).

**Table 5:** Standardized indirect statistical associations and total associations of psychological distress and subjective well-being with university dropout intention through satisfaction with education and academic burnout (*n* = 614)

Effect type	Pathway	β	95% CI	*p*
Indirect	PD → SE → UDI	−0.014	−0.034, 0.006	0.161
	PD → AB → UDI	0.289	0.198, 0.380	< 0.001
	PD → Total indirect	0.275	0.186, 0.364	< 0.001
	SWB → SE → UDI	0.006	−0.011, 0.024	0.472
	SWB → AB → UDI	−0.105	−0.187, − 0.024	0.011
	SWB → Total indirect	−0.099	−0.178, − 0.020	0.014
Total (direct + indirect)	PD → UDI	0.196	0.084, 0.308	0.001
	SWB → UDI	−0.052	−0.151, 0.048	0.311

*PD* psychological distress, *SWB* subjective well-being, *SE* satisfaction with education, *AB* academic burnout, *UDI* university dropout intention, β standardized regression coefficient, *CI* confidence interval

Overall, the hypothesis testing results indicated partial support for the proposed model. H1 was partially supported, as psychological distress and subjective well-being were not directly associated with university dropout intention but showed significant indirect statistical associations through academic burnout. H2 was supported, with academic burnout positively associated and satisfaction with education negatively associated with university dropout intention. H3 and H4 received partial support because only the indirect pathways involving academic burnout were statistically significant, whereas the indirect pathways involving satisfaction with education were not supported.

## Discussion

The present study examined the relationships among psychological distress, subjective well-being, academic burnout, satisfaction with education, and university dropout intention using a structural equation modeling approach. The model showed adequate fit and explained a substantial proportion of variance in university dropout intention, supporting its relevance within explanatory frameworks of university dropout intention and student withdrawal processes [23, 24]. Mental health variables were not directly associated with university dropout intention but showed significant indirect associations involving academic burnout, as reflected in significant indirect effects and non-significant direct effects. The explained variance was high for university dropout intention, moderate for academic burnout, and minimal for satisfaction with education, indicating differential explanatory capacity across endogenous variables. In line with contemporary conceptualizations, university dropout intention can be understood as a proximal cognitive indicator of withdrawal processes rather than a direct reflection of academic failure [4].

In line with these findings, the hypotheses received mixed support. H1 was not supported, as no significant direct associations were found between psychological distress or subjective well-being and university dropout intention. H2 was supported, given that academic burnout was positively associated and satisfaction with education was negatively associated with university dropout intention. H3 was partially supported, as academic burnout mediated the relationship between subjective well-being and university dropout intention, whereas satisfaction with education did not. H4 was also partially supported, since psychological distress showed a significant indirect effect on university dropout intention through academic burnout, but not through satisfaction with education.

The most salient finding of this study is the strong association between academic burnout and university dropout intention. This result is consistent with previous research identifying burnout as a construct consistently associated with academic disengagement and the decision to leave university [16, 17]. However, the magnitude of this association (β = 0.900) warrants cautious interpretation. Although it may reflect a substantive relationship, it could also indicate partial conceptual proximity between both constructs, as academic burnout captures aspects of exhaustion and disengagement that are theoretically close to university dropout intention. Nevertheless, the constructs were empirically distinguishable. Discriminant validity was supported by HTMT values below the recommended threshold, and the measurement models showed adequate factorial structure. The magnitude of this relationship indicates a strong statistical association between academic burnout and university dropout intention within a cross-sectional self-report model, although it should not be interpreted as evidence of a causal mechanism. One possible interpretation is that higher perceived academic overload and disengagement co-occur with greater dropout intention; however, longitudinal evidence is needed to clarify temporal ordering. This pattern of association may be conceptually interpreted, in light of Tinto’s integration model (1975) [24]—as reflecting academic disintegration that weakens students’ attachment to the institution and increases students’ intention to withdraw from higher education.

Regarding psychological distress, no statistically significant direct association with university dropout intention was observed; however, a significant indirect association emerged through academic burnout. This pattern is statistically compatible with an indirect association involving academic burnout, suggesting that symptoms of depression, anxiety, and stress co-occurred with higher university dropout intention through pathways represented by burnout within the proposed model. However, given the cross-sectional design, these findings should not be interpreted as evidence of temporal or causal mediation [44]. These findings align with previous studies indicating that the impact of psychological distress on academic outcomes tends to be channeled through intermediate variables such as burnout, engagement, or resilience [9, 10, 45]. In this context, the absence of direct association may reflect the distal nature of psychological distress in relation to specific academic decisions, whereas burnout was strongly associated with university dropout intention within the proposed model [19]. The differences observed between bivariate correlations and direct effects estimated in the SEM may reflect adjustment effects arising from the simultaneous inclusion of multiple factors and indirect pathways within the structural model. Therefore, these findings should not be interpreted as contradictory but rather as representing distinct levels of analysis. Moreover, suppression effects have been documented in structural models involving correlated constructs and may contribute to attenuating or eliminating apparent direct associations [17].

Similarly, subjective well-being did not show a significant direct association with university dropout intention but exhibited a statistically significant indirect association involving academic burnout within the proposed model. This finding reinforces the notion that well-being may operate as a psychological resource associated with lower academic burnout, rather than directly influencing university dropout intention. This result is consistent with evidence linking subjective well-being to better academic adjustment and lower levels of exhaustion [11, 13, 17], as well as with recent studies that have integrated both positive and negative indicators of mental health within structural models [46, 47].

A notable finding of the model was the significant negative association between subjective well-being and psychological distress, indicating that although these constructs are inversely related, they are not simply opposite ends of the same continuum. This result supports the dual-continuum model of mental health, which posits that subjective well-being and distress are related but conceptually distinct dimensions [15]. This pattern has important implications for understanding university dropout, as it suggests that reducing distress alone does not necessarily lead to increased subjective well-being, highlighting the need to address both components in a complementary manner. Moreover, the moderate magnitude of this association suggests that subjective well-being may function as a relatively independent psychological resource capable of buffering the impact of distress on academic experiences, particularly academic burnout.

In turn, satisfaction with education showed a significant negative association with university dropout intention, consistent with previous literature suggesting that students with less favorable educational experiences may be more likely to consider leaving higher education [20, 21]. However, its mediating role between mental health variables and university dropout intention was not supported, and the explained variance for this construct was minimal (R² = 0.011). Therefore, these findings should be interpreted cautiously. Satisfaction with education may operate as a broader evaluative construct influenced by institutional and contextual conditions rather than functioning as a proximal psychological mechanism within the proposed model. Its low explained variance further suggests that additional determinants not included in the model—such as perceived program quality, faculty–student interaction, institutional support, or broader educational environment conditions—may contribute substantially to students’ educational satisfaction.

Overall, the results support a model in which mental health variables are statistically associated with university dropout intention through pathways involving academic experiences, particularly academic burnout, within a cross-sectional framework. This finding provides empirical support for integrative models of university dropout [23, 24]. Furthermore, it advances the understanding of processes underlying university dropout intention by simultaneously integrating negative (psychological distress) and positive (subjective well-being) indicators, demonstrating that both were associated with a common pattern involving academic burnout. Thus, the study reinforces the relevance of academic burnout as a construct strongly associated with university dropout intention and provides evidence compatible with an indirect statistical role within the proposed model.

This study also contributes relevant evidence from Latin American contexts, where models integrating psychological and academic variables in the explanation of university dropout intention remain limited [30, 36]. Particularly in contexts marked by structural inequalities and heterogeneous educational conditions, understanding the role of mental health in processes related to student persistence and university dropout intention is critical. Accordingly, these findings help contextualize theoretical models developed in global settings by providing empirical evidence from a Colombian university population.

These findings may contribute to informing approaches aimed at supporting students’ academic experiences and psychological subjective well-being. In particular, institutional considerations may include procedures aimed at identifying students experiencing elevated levels of academic burnout, strengthening academic advising and mentoring initiatives, periodically reviewing perceived academic workload and available support resources, and facilitating access to referral pathways for psychological support services when appropriate. Although causal inferences cannot be drawn, the present findings may help generate hypotheses and guide institutional planning intended to support students’ academic experiences and psychological well-being.

However, the findings should be interpreted in light of several limitations. First, the cross-sectional design does not allow for causal inferences. Accordingly, the strong association between academic burnout and university dropout intention should not be interpreted as evidence of a demonstrated causal mechanism or intervention target. In addition, the estimation of indirect association in cross-sectional models may lead to an overestimation of mediation, so longitudinal studies are needed to more accurately examine the directionality and temporal stability of these effects.

Second, although stratified sampling was used, the study was conducted in a single institution, which may limit the generalizability of the findings to other university settings. Furthermore, the predominance of students from Health Sciences programs reflected the academic composition of the participating university, and caution is therefore warranted when extrapolating these findings to students from other disciplinary contexts. Future research should include multicenter samples and populations with greater sociocultural diversity to strengthen external validity. Because the total number of invited students was not tracked after additional invitations were issued to compensate for non-response, an exact response rate could not be calculated. Consequently, the potential impact of non-response bias cannot be fully excluded.

Third, all variables were assessed using self-report instruments collected at a single time point, which may introduce bias related to common method variance and social desirability. Although Harman’s single-factor test suggested that common method bias was unlikely to substantially affect the findings, the use of self-report measures collected at a single time point means that some degree of shared method variance cannot be completely excluded. Future studies should incorporate multimethod approaches, including objective academic indicators, institutional dropout records, and complementary data sources, to improve the triangulation of findings.

Finally, although the model included theoretically relevant psychological and academic variables, it did not consider other individual, family, institutional, sociodemographic, or academic factors that may influence university dropout intention. In particular, sociodemographic and academic covariates such as gender, GPA, employment status, and time at university were not explicitly incorporated into the structural model, as the SEM was theoretically specified to examine relationships among latent psychological and academic constructs and their mediating mechanisms. These characteristics may nevertheless contribute to variability in university dropout intention and related academic experiences. Future studies should expand the model by incorporating additional variables and evaluating the robustness of the proposed relationships through expanded or sensitivity analyses, as well as testing model stability across different cultural and institutional contexts to deepen understanding of student persistence processes.

## Conclusion

This study provides empirical evidence on the psychological and academic factors associated with university dropout intention. Academic burnout emerged as the strongest correlate of university dropout intention within the proposed model. Psychological distress and subjective well-being were indirectly associated with university dropout intention through statistical pathways involving academic burnout. In contrast, satisfaction with education, despite being negatively associated with university dropout intention, did not participate in the indirect statistical associations between mental health indicators and dropout intention. These findings should be interpreted as associative rather than causal and may contribute to informing future longitudinal research and institutional approaches related to students’ mental health and academic experiences. These findings contribute to the growing body of evidence on university dropout intention in Latin American contexts.

## Data Availability

The datasets generated during the current study are not publicly available due to protect the participants’ privacy but are available from the corresponding author on reasonable request.
